# Supplementary material for: A pathway towards decentralized studies of radioactive post-lead elements and their applications in beyond standard model physics
Source: Nat Commun. 2026 Sep 10;17:10681. doi: 10.1038/s41467-026-77082-2 (PMC13655031; doi:10.1038/s41467-026-77082-2)
Supplement: Supplementary file 1 [file 41467_2026_77082_MOESM1_ESM.pdf]

Supplementary Information for: A pathway towards decentralized studies of radioactive post-lead elements and their applications in beyond standard model physics

Moritz Pascal Reiter[1*], Kriti Mahajan[2,3], Meetika Narang[4,5], Carsten Zülch[6], Timo Dickel[2,4], Daler Amanbayev[2,4], Robert Berger[6], Julian Bergmann[2], Agnieszka Bukowicka[1], Mariam Fadel[6], Tayemar Fowler-Davies[1,4], Zhuang Ge[4], Simeon Gloeckner[4], Gabriella Kripkó-Koncz[1,2], Nasser Kalantar-Nayestanaki[5], Cameron Merron[1], David J. Morrissey[4,7], Wolfgang R. Plaß[2,4], Christoph Scheidenberger[2,3,4], Makar Simonov[2,3], Nazarena Tortorelli[4,8], Jiajun Yu[4], Alexandra Zadvornaya[1,2], Jianwei Zhao[4]

[1]School of Physics and Astronomy, University of Edinburgh, Edinburgh, EH9 3FD, United Kingdom.
[2]II. Physikalisches Institut, Justus-Liebig Universitaet Giessen, Giessen, 35392, Germany.
[3]Helmholtz Research Academy Hesse for FAIR (HFHF), GSI Helmholtz Center for Heavy Ion Research, Campus Gießen, Giessen, 35392, Germany.
[4]GSI Helmholtz Centre for Heavy Ion Research GmbH, Darmstadt, 64278, Germany.
[5]Nuclear Energy Group ESRIG, University of Groningen, Groningen, 9747 AA, The Netherlands.
[6]Department of Chemistry, Philipps-Universität Marburg, Marburg, 35032, Germany.
[7]Department of Chemistry, Michigan State University, East Lansing, MI 48824, United States.
[8]Faculty of Physics, Ludwig-Maximilians-Universität München, Garching, 85748, Germany.

*Corresponding author(s). E-mail(s): mreiter@ed.ac.uk;

Supplementary Note 1: Validation of quantum-chemical calculations

Supplementary Table 1 and Supplementary Table 2, benchmark the electronic structure approach used in this work (RECP-ROHF-UCCSD(T)) against available literature data for ionization energies, electron affinities and selected dissociation energies of Pb-, Po- and Ra-containing species. For Ra and RaF, the computed ionization energies agree closely with experiment, supporting the accuracy of the method for the key charge states involved in the fluorination reactions. For Pb and Po, larger deviations are observed for some ionization energies, consistent with the more pronounced multi-reference character of their low-lying electronic states and remaining spin–orbit effects not explicitly included in the present treatment. For Po, the electron affinity is reproduced within the range of reported theoretical values. Overall, these benchmarks support the use of the calculations to interpret the experimentally observed reaction channels and trends, while absolute energies for Pb/Po should be viewed with correspondingly larger uncertainty.

Supplementary Table 1 Electronic ionization energies (IE), electron affinities (EA) and dissociation energies (DE) of the various F, OH and $SF_{5,6}$ species computed adiabatically on the level of ROHF-UCCSD(T) with and without frozen orbitals are given with literature values if present.

	Lit.		ROHF-UCCSD(T)-frozen-core	ROHF-UCCSD(T)
IE(F)	17.4	[1]	17.4	17.4
EA(F)	3.40	[2]	3.38	3.39
IE(OH)	13.0	[3]	13.0	13.0
EA(OH)	1.83	[4]	1.79	1.80
DE($H_2O \rightarrow H + OH$)	5.10	[5]	5.42	5.44
IE(SF_6)	15.1	[6]	16.0	16.0
IE(SF_5)	9.60	[7]	9.84	9.78
DE($SF_6 \rightarrow SF_5^+ + F^-$)			11.1	11.2
DE($SF_6 \rightarrow SF_5 + F$)			4.63	4.82

Supplementary Table 2 Electronic ionization energies (IE), electron affinities (EA) and dissociation energies (DE) of relevant systems including Pb, Po or Ra computed adiabatically on the level of ROHF-UCCSD(T) are given with selected experimental (Lit. Exp.) and theoretical (Lit. Theo.) data from literature.

	Lit. Exp.	Lit. Theo.	ROHF-UCCSD(T)
IE(Pb)	7.42 [8]	7.04 [9]	6.87
IE(Pb^+)	15.0 [10]	14.3 [9]	13.4
IE(PbF)	7.55 [11]	7.44 [12]	6.72
DE($PbF^+ \rightarrow Pb + F^+$)		15.1 [13]	13.0
DE($PbF^+ \rightarrow Pb^+ + F$)		4.72 [13]	2.54
DE($PbOH^+ \rightarrow Pb + OH^+$)			8.10
DE($PbOH^+ \rightarrow Pb^+ + OH$)			1.98
IE(Po)	8.42 [14]	8.43 [15]	8.08
IE(Po [4z])	8.42 [14]	8.43 [15]	8.18
IE(Po [5z])	8.42 [14]	8.43 [15]	8.22
IE(Po^+)		19.4 [16]	17.8
IE(Po^+ [4z])		19.4 [16]	17.9
IE(Po^+ [5z])		19.4 [16]	17.9
EA(Po)		1.469-1.9 [15, 17] [a]	1.79
EA(Po [4z])		1.469-1.9 [15, 17] [a]	1.88
EA(Po [5z])		1.469-1.9 [15, 17] [a]	1.91
IE(PoF)			8.50
IE(PoF^+)			17.3
IE(PoF^+ [4z])			17.3
DE($PoF^+ \rightarrow Po + F^+$)			12.0
DE($PoF^+ \rightarrow Po^+ + F$)			2.77
DE($PoOH^+ \rightarrow Po + OH^+$)			6.66
DE($PoOH^+ \rightarrow Po^+ + OH$)			1.76
IE(Ra)	5.28 [18]	5.29 [19]	5.23
IE(Ra^+)	10.1 [20]	10.1 [21]	10.1
IE(RaF)	4.97 [22]	4.97 [22]	4.95
DE($RaF^+ \rightarrow Ra + F^+$)			17.9
DE($RaF^+ \rightarrow Ra^+ + F$)		5.59 [23]	5.74
DE($RaOH^+ \rightarrow Ra + OH^+$)			12.4
DE($RaOH^+ \rightarrow Ra^+ + OH$)			4.59

[a]Used ECP was assessed against EA(Po)=1.90 eV [17]. A more recent computation on the level of 4c+CCSD(T)+Breit+QED predicts 1.47 eV [15].

Supplementary References

References

[1] Edlén, B. On the identification of Ar x and Ar xiv in the solar corona and the origin of the unidentified coronal lines. *Sol. Phys.* **9**, 439–445 (1969). URL https://doi.org/10.1007/BF02391668.

[2] Blondel, C., Delsart, C. & Goldfarb, F. Electron spectrometry at the μeV level and the electron affinities of Si and F. *J. Phys. B: Atom. Mol. Opt. Phys.* **34**, L281–L288 (2001). URL http://stacks.iop.org/0953-4075/34/i=9/a=101.

[3] Wiedmann, R. T., Tonkyn, R. G., White, M. G., Wang, K. & McKoy, V. Rotationally resolved threshold photoelectron spectra of OH and OD. *J. Chem. Phys.* **97**, 768–772 (1992). URL https://doi.org/10.1063/1.463179.

[4] Smith, J. R., Kim, J. B. & Lineberger, W. C. High-resolution threshold photodetachment spectroscopy of oh-. *Phys. Rev. A* **55**, 2036–2043 (1997). URL https://link.aps.org/doi/10.1103/PhysRevA.55.2036.

[5] Ruscic, B. *et al.* On the enthalpy of formation of hydroxyl radical and gas-phase bond dissociation energies of water and hydroxyl. *J. Chem. Phys. A* **106**, 2727–2747 (2002). URL https://doi.org/10.1021/jp013909s.

[6] Yencha, A. *et al.* Threshold photoelectron spectroscopy of SF_6. *Chem. Phys.* **216**, 227–241 (1997). URL https://www.sciencedirect.com/science/article/pii/S0301010496003722.

[7] Fisher, E. R., Kickel, B. L. & Armentrout, P. B. Collision-induced dissociation and charge transfer reactions of SF_x^+ (x=1–5): Thermochemistry of sulfur fluoride ions and neutrals. *J. Chem. Phys.* **97**, 4859–4870 (1992). URL https://doi.org/10.1063/1.463840.

[8] Dembczyński, J., Stachowska, E., Wilson, M., Buch, P. & Ertmer, W. Measurement and interpretation of the odd-parity levels of Pb I. *Phys. Rev. A* **49**, 745–754 (1994). URL https://link.aps.org/doi/10.1103/PhysRevA.49.745.

[9] Yu, Y. J., Dong, C. Z., Li, J. G. & Fricke, B. The excitation energies, ionization potentials, and oscillator strengths of neutral and ionized species of Uuq (Z=114) and the homolog elements Ge, Sn, and Pb. *J. Chem. Phys.* **128**, 124316 (2008). URL https://doi.org/10.1063/1.2838985.

[10] Hanni, M. E., Keele, J. A., Lundeen, S. R., Fehrenbach, C. W. & Sturrus, W. G. Polarizabilities of pb2+ and pb4+ and ionization energies of pb+ and pb3+ from spectroscopy of high-ls rydberg states of pb+ and pb3+. *Phys. Rev. A* **81**, 042512 (2010). URL https://link.aps.org/doi/10.1103/PhysRevA.81.042512.

[11] Sivakumar, P., McRaven, C. P., Combs, D., Shafer-Ray, N. E. & Ezhov, V. State-selective detection of the PbF molecule by doubly resonant multiphoton ionization. *Phys. Rev. A* **77**, 062508 (2008). URL https://link.aps.org/doi/10.1103/PhysRevA.77.062508.

[12] Yamamoto, S. & Tatewaki, H. Excited states of PbF: A four-component relativistic study. *J. Chem. Phys.* **132**, 054303 (2010). URL https://doi.org/10.1063/1.3298583.

[13] Ghosh, S. & Ghosh, T. Spectroscopic properties and bond dissociation energies of PbX, $PbX^{\pm}$, PbX_2 and $PbX_2^{\pm}$ (X=F, Cl, Br, I). *J. Indian Chem. Soc.* **99**, 100310 (2022). URL https://www.sciencedirect.com/science/article/pii/S0019452221003101.

[14] Fink, D. *et al.* Determination of the first ionization energy of polonium by resonance ionization spectroscopy – part ii: Measurement of odd-parity rydberg states at cern–isolde. *Spectrochim. Acta B* **151**, 72–82 (2019). URL https://www.sciencedirect.com/science/article/pii/S0584854718302453.

[15] Borschevsky, A., Pašteka, L. F., Pershina, V., Eliav, E. & Kaldor, U. Ionization potentials and electron affinities of the superheavy elements 115–117 and their sixth-row homologues Bi, Po, and At. *Phys. Rev. A* **91**, 020501 (2015). URL https://link.aps.org/doi/10.1103/PhysRevA.91.020501.

[16] Finkelnburg, W. & Humbach, W. Ionisierungsenergien von Atomen und Atomionen. *Naturwissenschaften* **42**, 35–37 (1955). URL https://doi.org/10.1007/BF00621525.

[17] Peterson, K. A., Figgen, D., Goll, E., Stoll, H. & Dolg, M. Systematically convergent basis sets with relativistic pseudopotentials. ii. small-core pseudopotentials and correlation consistent basis sets for the post-d group 16–18 elements. *J. Chem. Phys.* **119**, 11113–11123 (2003). URL https://doi.org/10.1063/1.1622924.

[18] Armstrong, J. A., Wynne, J. J. & Tomkins, F. S. Bound, 7snp $^1P_1^0$ series in Ra I: measurements and predictions. *J. Phys. B: At. Mol. Opt. Phys.* **13**, L133 (1980). URL https://dx.doi.org/10.1088/0022-3700/13/5/002.

[19] Ginges, J. S. M. & Dzuba, V. A. Spectra of barium, radium, and element 120: Application of the combined correlation-potential, singles-doubles, and configuration-interaction ab initio methods. *Phys. Rev. A* **91**, 042505 (2015). URL https://link.aps.org/doi/10.1103/PhysRevA.91.042505.

[20] Dammalapati, U., Jungmann, K. & Willmann, L. Compilation of spectroscopic data of radium (Ra I and Ra II). *J. Phys. Chem. Ref. Data* **45**, 013101 (2016). URL https://doi.org/10.1063/1.4940416.

[21] Skripnikov, L. V. Approaching meV level for transition energies in the radium monofluoride molecule RaF and radium cation Ra^+ by including quantum-electrodynamics effects. *J. Chem. Phys.* **154**, 201101 (2021). URL https://doi.org/10.1063/5.0053659.

[22] Wilkins, S. *et al.* Ionization potential of radium monofluoride. *arXiv preprint arXiv:2408.14673* (2024). URL https://arxiv.org/abs/2408.14673.

[23] Isaev, T. A., Hoekstra, S., Willmann, L. & Berger, R. Ion neutralisation mass-spectrometry route to radium monofluoride (RaF). *ArXiv e-prints* (2013). URL https://doi.org/10.48550/arXiv.1310.1511.
