# Supplementary material for: A pathway towards decentralized studies of radioactive post-lead elements and their applications in beyond standard model physics
Source: Nat Commun. 2026 Sep 10;17:10681. doi: 10.1038/s41467-026-77082-2 (PMC13655031; doi:10.1038/s41467-026-77082-2)
Supplement: Supplementary file 2 [file 41467_2026_77082_MOESM2_ESM.pdf]

nature portfolio

# Peer Review File

# A pathway towards decentralized studies of radioactive post-lead elements and their applications in beyond standard model physics

Corresponding Author: Dr Moritz Reiter

**This file contains all reviewer reports in order by version, followed by all author rebuttals in order by version.**

Version 0:

Reviewer comments:

Reviewer #1

(Remarks to the Author)
The manuscript describes an offline (accelerator-independent) method to produce and identify heavy radioactive molecular ions. Recoiling α-decay daughters are harvested in a cryogenic stopping cell, transferred into an RFQ region where controlled ion–molecule reactions are initiated, and the resulting products are identified with broadband MR-TOF mass spectrometry. The central experimental result is the rapid and highly efficient formation of $RaF^+$ from $Ra^{2+}$ + $SF_6$, approaching complete conversion on ~10 ms timescales (Fig. 2), together with demonstrations of $PoF^+$ and $PbF^+$ production exhibiting strong charge-state selectivity (Figs. 3–4; Table 1). Quantum-chemistry calculations are used to interpret the observed charge-state dependence and to assess possible secondary reaction pathways (Fig. 5; Tables 2–4).

The subject is timely and could be impactful for "decentralized" radioactive-molecule research. However, several quantitative conclusions (in particular the extracted rate coefficients, conversion efficiencies, and impurity-driven chemistry) are presently limited by diagnostics—an issue that the authors also acknowledge.

In my view, the overall experimental concept is clearly novel, even if some individual elements have prior precedent. Importantly, the approach is likely to be reproducible in university-scale laboratories aiming at molecular spectroscopy and, ultimately, searches for symmetry-violating effects. The method also appears readily generalizable to other short-lived isotopes that can be generated using recoil sources.

I would support publication in *Nature Communications*, provided the authors address the points below. I start with the main major comment, while all others are more minor.

Major:
* While I appreciate that additional measurement time is costly, I would strongly encourage repeating the key kinetics measurements once the improved diagnostics are available. This would elevate the paper from an important methodological demonstration to a quantitatively strong kinetics dataset. Since a central selling point is that the method no longer depends on accelerator access, thus enabling sustained and systematic measurements, it would be unfortunate if this is not reflected in the precision of the reported rate coefficients. I appreciate that this might cause significant delays to the publication of this work, but I believe it would be well justified.

Minor:
* The manuscript contains a number of typographical errors. There are also several long, comma-heavy sentences that would benefit from rewriting for clarity.
* The authors state that recoil products are extracted in singly, doubly, and triply charged states. What fraction is expected to be produced (or extracted) in the neutral charge state?
* Please clarify the role of cryogenic operation of the stopping cell. Is the low temperature essential beyond maintaining gas purity (e.g., by suppressing impurities/outgassing), or does the temperature itself materially affect stopping, extraction, or chemistry? This matters for how straightforward implementation would be in typical university laboratories.
* I recommend stating the source activity explicitly in the main text, and giving representative observed count rates for Ra in the different charge states. This would enable an efficiency estimate: what fraction of recoil daughters are stopped,

transported, and ultimately detected? Where does the dominant loss occur, and is there a clear path to improving it?
* Closely related: a short discussion of realistic scaling prospects would be valuable for readers assessing what future measurements might be feasible. For example, recoil sources cannot be arbitrarily thick; additional limitations may arise from space-charge effects or ion transport saturation.
* There appears to be an inconsistency in the quoted $SF_6$ pressure: the text cites ~$8\times10^{-5}$ mbar for some measurements, whereas the table caption gives $9(4)\times10^{-3}$ mbar. Perhaps this refers to different conditions during those measurements. In any case, how are these pressures determined, and how is the quoted uncertainty obtained? At present the uncertainty appears somewhat ad hoc, yet it is central to the comparison with other alkaline-earth systems. I would also recommend the authors provide more detail on the fitting approach (least squares? likelihood fits?) and that the explicit rate equation model is also given in the methods section, for completeness.

Reviewer #2

(Remarks to the Author)
Referee report.
Nature Communications manuscript NCOMMS-25-102894

A pathway towards decentralized studies of radioactive post-lead elements and their applications in beyond standard model physics
P.M. Reiter et al

The manuscript by Reiter and colleagues presents an efficient method to harvest long-lived radioactive isotopes for research without the general requirement of a large-scale accelerator facility. Of particular interest in this work is the production of radioactive molecules, which have undergone increasing interest from different fields in recent years (e.g., for fundamental symmetries, astrophysics, nuclear structure and quantum chemistry). This interest in part has been due to advances in the ability to create, cool and control molecules, and to wider opportunities for production of radioactive molecules at RIB facilities.
The approach presented here would potentially be accessible for smaller (university-scale) laboratories. In brief, the authors employ a cryogenic gas-filled stopping cell as an environment for harvesting radioactive ions from a 228Th source. A combination of DC and RF fields are used to extract the ions from the cell into a radiofrequency quadrupole beam line. Operation of this device as a low-resolution mass separator allows for charge state selection of the ions which, in a high-purity environment, typically survive as singly- or doubly charged ions (albeit higher charge states in some cases are possible). Elements in the beam line are tuned to accumulate ions which are then exposed to controlled trace amounts of reactive gas, in this case SF6, to explore fluorination reactions with the different radioactive decay daughters (Ra, Po and Pb ions) extracted from the gas cell.
As the authors discuss, the present work focuses on the formation of RaF given its importance as a candidate for searches of physics beyond the standard model. To quantify the underlying gas-phase reaction rates between Ra+/2+ and SF6, as well as with other nuclei in the decay chain, sensitive identification of the ion species is necessary. The chosen method is high-resolution mass spectrometry in which unambiguous mass identification is demonstrated through resulting time-of-flight mass spectra. Reiter and colleagues use the mass fingerprint to study the evolution of the different species under different experimental conditions: number of revolutions in the MR-TOF device, selection of different charge states and the introduction – or otherwise – of SF6 gas. By identifying the relevant species and the relative intensities of the mass peaks, the authors demonstrate a clear sensitivity to reaction processes as a function of accumulation time in the RFQ beam line, with reaction time constants extracted from simple exponential decay and/or growth curves. This information is combined with the measured SF6 concentration to determine fluoridation reaction rates, with data for Ra obtained for the first time. The authors compare their values with literature measurements of lighter alkaline earth metal ions, with the new results found to be in general agreement.
Finally, the experimental observations are interpreted using quantum-chemistry calculations, starting with restricted open shell Hartree-Fock calculations as a reference, and subsequent unrestricted coupled-cluster computations. These methods were assessed by calculating ionization energies, electron affinities and dissociation energies for the different atoms and molecules of interest (expounded upon in the Methods), and benchmarking against (limited) experimental and theoretical values from literature. The experimentally observed reaction pathways as elucidated from the mass spectra are then compared with the computed reaction energies which may be endothermic or exothermic. Those predicted to be exothermic in nature were observed in the mass spectra.
In summary, the article is well written and accessible to the general reader. The work has been put into context with efforts to develop new platforms for fundamental physics studies, as well as with the long-standing field of gas-phase ion chemistry. The experimental methodology is robust and sufficiently well explained, with suitable use of figures and tables. The analysis of the mass spectra and the analytical approach to determine reaction rates is valid. The additional inclusion of quantum-chemistry calculations provides added value to the experimental data, supporting the understanding of the reaction pathways open to certain elements and molecules, and validating the non-observation of others which can be explained through the computed reaction energies. Given the novelty of the methodologies with the clear potential for future harvesting of other long-lived actinide species as well as further gas-phase ion chemistry studies (both of other elements as well as trace gases), I believe this article is suitable for the Nature Communications audience. I have only a few minor comments for the author's consideration.

1. Given the use of the 228Th source in this work and the variety of accessible decay products, it would be helpful to include a figure showing the basic decay scheme, decay mode and half-lives. Such a figure could be considered for the Methods section 6.1. This also gives context for the reader to understand that all species are extracted from the gas cell within the

extraction time of the cell – which I don't recall being explicitly mentioned however could be included in the Methods.
2. Although the authors stress several times that the methods employed allow for future "decentralized studies" without the need for accelerators and/or reactors, one might argue that the cryogenic stopping cell employed in this work is perhaps not the simplest of devices for a university laboratory. Given the small recoil energy in alpha decay of heavy elements, it would be worthwhile adding a sentence at the end of Section 2 to stress that smaller scale gas cells would be applicable to realize extraction of recoil ions as described in this work.
3. Abstract line 61, "…further supported by…"
4. Figure 1, correct "accumulation" in the label pointing towards the reaction region.
5. I suggest reordering Figures 2 and 3, given Fig. 3 is referenced first in the text.
6. A sentence or appropriate reference could be added to indicate what kind of peak fit is used when extracting the intensities of the ion species which are used in the gas-phase chemistry analysis.
7. Page 10, line 445, "…or release of the reactions studies."
8. Figure 5 and Table 2. I might be mistaken, but can you clarify the singly charged reaction pathway between $MF^+$ and $H_2O$? In Fig. 5 it appears that for Ra and Po the energy for both is negative (see right hand side of the figure), while in the last row of Table 2 they are positive (values 0.45 eV and 0.323 eV, respectively).
9. Page 12, lines 512-513, "…as a potential loss channel, for example HF formation…" Line 532, "Despite the larger uncertainty…"
10. Table 3. It was not immediately clear to me why m(Ca) = 44u was used, as mentioned in the caption. Naively perhaps I would think 40 u would have been chosen.
11. Reference 31 seems to be missing information. Is this a thesis? If so, consider adding the relevant institution.

Reviewer #3

(Remarks to the Author)
The manuscript "A pathway towards decentralized studies of radioactive post-lead elements and their applications in beyond standard model physics" demonstrates an important step toward the expansion of off-line capabilities in the study of radioactive molecules, and provides measurements which are of interest on their own. Given the rapid increase in interest in the precision study of radioactive atoms and molecules, further tools which enable their study are likely to be widely adopted, especially outside of the few large facilities with direct access to radioactive beams. Both the methods and results appear to be sound, are likely of broad interest, and will likely be impactful to a wide community. I believe the manuscript to be suitable for publication in Nature Communications, though I recommend additional discussion about other approaches to decentralized studies.

As written, a non-expert reader would probably conclude that this is the first demonstration of a decentralized approach, though that is not the case. There are many such avenues being pursued, but two relevant ones have been experimentally demonstrated: trapped radium atomic and molecular ions at UCSB, and cryogenically-cooled neutral radium molecules at Caltech. The manuscript offers some distinct advantages over these other two methods, but there are also some important similarities.

The UCSB group has demonstrated work with multiple molecular species formed through controlled chemical reactions [reference 61] and multiple radium isotopes, including two short-lived isotopes Ra-224 [reference 62] and Ra-225 [2407.14721, unreferenced]. The UCSB Ra-224 work used decays from Th-228, as in this manuscript. However, the manuscript work uses a gas cell, which makes the approach applicable to a wider range of studies outside of ion trapping, to which the UCSB approach (as currently implemented) is largely limited.

The Caltech group has demonstrated cryogenic formation of multiple radium-containing species including polyatomics via laser-controlled chemistry, and has shown high resolution optical spectra [2508.08368, not referenced]. However, the manuscript approach does not rely on any sort of handling of the radioactive species of interest, and is therefore well-suited to short-lived species as discussed in the caption of Fig. 1.

My specific recommendation is that there be some discussion in the introduction, on the level of a few sentences, which compare and contrast with these different approaches, and perhaps a brief mention in the conclusion as well. The UCSB results are mentioned very briefly in passing, but should be discussed more deliberately; the Caltech work is not referenced. Again, these studies do not detract from the impact or novelty of the present result, as they all provide a distinct set of advantages.

Version 1:

Reviewer comments:

Reviewer #1

(Remarks to the Author)
I think the authors for the extensive changes to the manuscript. I belive they nicely improve the quality and usefulness of the paper. I have no further comments.

Reviewer #2

(Remarks to the Author)

I thank the authors for this revised version of the submitted manuscript and their detailed responses to my suggestions, as well as responses to the remarks made by the other two reviewers. The minor corrections have been done to my satisfaction, and the context has been sufficiently adjusted to allow the reader to better appreciate the scope for scaling such experiments to smaller volume gas cells (potentially without cryogenic operation), which may be more in line with possible university laboratory-scale efforts.

This is a timely and novel addition to wider efforts to implement techniques for the study of radioactive molecules, with the potential to explore a wide-range of molecules in the future which, combined with the theoretical efforts, will hopefully lead to tailor-made systems for future fundamental symmetry tests.

I have no further suggestions and recommend that the article now be accepted for publication.

Reviewer #3

(Remarks to the Author)
I believe that the manuscript is suitable for publication as-is. Very nice work.

**Open Access** This Peer Review File is licensed under a Creative Commons Attribution 4.0 International License, which permits use, sharing, adaptation, distribution and reproduction in any medium or format, as long as you give appropriate credit to the original author(s) and the source, provide a link to the Creative Commons license, and indicate if changes were made.
In cases where reviewers are anonymous, credit should be given to 'Anonymous Referee' and the source.
The images or other third party material in this Peer Review File are included in the article's Creative Commons license, unless indicated otherwise in a credit line to the material. If material is not included in the article's Creative Commons license and your intended use is not permitted by statutory regulation or exceeds the permitted use, you will need to obtain permission directly from the copyright holder.
To view a copy of this license, visit https://creativecommons.org/licenses/by/4.0/

**Response to REVIEWER COMMENTS**

We thank the Editor and all three reviewers for their careful reading of the manuscript and for their constructive and insightful comments. We are grateful for the positive assessment of the overall significance and methodological advance of the work, as well as for the detailed suggestions that helped us improve the clarity, scope, and presentation of the manuscript. In response, we have revised the paper substantially, including clarifying the role of the cryogenic stopping cell, improving the treatment and presentation of the gas-pressure diagnostics and kinetics analysis, adding discussion of complementary decentralized approaches, and correcting a number of typographical and presentation issues. Below, we provide a point-by-point response to all comments and indicate how the manuscript has been revised accordingly.

Reviewer #1 (Remarks to the Author):

The manuscript describes an offline (accelerator-independent) method to produce and identify heavy radioactive molecular ions. Recoiling α-decay daughters are harvested in a cryogenic stopping cell, transferred into an RFQ region where controlled ion–molecule reactions are initiated, and the resulting products are identified with broadband MR-TOF mass spectrometry. The central experimental result is the rapid and highly efficient formation of $RaF^+$ from $Ra^{2+}$ + $SF_6$, approaching complete conversion on ~10 ms timescales (Fig. 2), together with demonstrations of $PoF^+$ and $PbF^+$ production exhibiting strong charge-state selectivity (Figs. 3–4; Table 1). Quantum-chemistry calculations are used to interpret the observed charge-state dependence and to assess possible secondary reaction pathways (Fig. 5; Tables 2–4).

The subject is timely and could be impactful for “decentralized” radioactive-molecule research. However, several quantitative conclusions (in particular the extracted rate coefficients, conversion efficiencies, and impurity-driven chemistry) are presently limited by diagnostics—an issue that the authors also acknowledge.

In my view, the overall experimental concept is clearly novel, even if some individual elements have prior precedent. Importantly, the approach is likely to be reproducible in university-scale laboratories aiming at molecular spectroscopy and, ultimately, searches for symmetry-violating effects. The method also appears readily generalizable to other short-lived isotopes that can be generated using recoil sources.

I would support publication in *Nature Communications*, provided the authors address the points below. I start with the main major comment, while all others are more minor.

We thank Reviewer #1 for the careful and constructive assessment of the manuscript, and in particular for highlighting both the strengths of the methodological advance and the present limitations of the quantitative diagnostics. The comments were very helpful in sharpening the presentation and clarifying the scope of the kinetics analysis.

Major:

* While I appreciate that additional measurement time is costly, I would strongly encourage repeating the key kinetics measurements once the improved diagnostics are available. This would elevate the paper from an important methodological demonstration to a quantitatively strong

kinetics dataset. Since a central selling point is that the method no longer depends on accelerator access, thus enabling sustained and systematic measurements, it would be unfortunate if this is not reflected in the precision of the reported rate coefficients. I appreciate that this might cause significant delays to the publication of this work, but I believe it would be well justified.

Response:

We thank the reviewer for this thoughtful comment and agree that improved in-situ gas diagnostics would strengthen the absolute precision of the reported rate coefficients. We also agree that, in an ideal scenario, repeating the key kinetics measurements with the upgraded diagnostics would further elevate the kinetics aspect of the work. At the same time, performing a new dedicated measurement campaign is, unfortunately, not feasible within a realistic revision timescale, because the specific instrument installed at GSI has since been reconfigured for scheduled online experiments at this facility and returning to the offline recoil-harvesting configuration required for the present study would require substantial additional preparation and long-term scheduling. This was precisely one of the main motivations for this proof-of-principle study that demonstrates the pathway to future decentralized studies and showcases their potential, once such installations operate at university scale.

In the revised manuscript, we have therefore addressed this point by substantially improving the transparency and scope of the kinetics analysis rather than by repeating the measurements at this stage. In particular, we now explicitly distinguish the helium buffer-gas pressure from the $SF_6$ partial pressure, clarify how the $SF_6$ pressure was determined and its uncertainty, state that this uncertainty dominates the absolute uncertainty of the rate coefficients, and make the fitting procedure and effective first-order reaction model explicit in the Methods. We have also adjusted the presentation so that the kinetics results are framed appropriately as a semi-quantitative proof-of-principle demonstration within the present diagnostic limitations.

We emphasize that the central conclusions of the manuscript do not depend on sub-10% precision in the absolute rate coefficients. The key findings that remain robust are the efficient and fast formation of radioactive monofluorides, the clear charge-state selectivity of the reactions, the unambiguous mass-resolved identification of precursor and product ions, and the demonstration that the full scheme operates on timescales compatible with short-lived isotopes. We agree that improved diagnostics will be highly valuable for future dedicated kinetics measurements, and we now make this future direction clearer in the revised manuscript.

Minor:

* The manuscript contains a number of typographical errors. There are also several long, comma-heavy sentences that would benefit from rewriting for clarity.

Response:

We thank the reviewer for pointing this out. We have carefully revised the text throughout and hope that the readability and clarity have been improved.

* The authors state that recoil products are extracted in singly, doubly, and triply charged states. What fraction is expected to be produced (or extracted) in the neutral charge state?

Response:

We thank the reviewer for this helpful question. The present experiment is sensitive only to the charged fraction of the recoil products, since neutral species are not transported electrostatically through the stopping cell and RFQ beam line. Based on independent detector measurements within the setup (using alpha spectroscopy and past records) , we estimate the stopping-cell extraction

efficiency for the charged recoil-ion beam to be of order 20-30% (see further break down including charge state distribution in the response below). The remaining neutral fraction of recoils does not contribute to the transported ion beam.

* Please clarify the role of cryogenic operation of the stopping cell. Is the low temperature essential beyond maintaining gas purity (e.g., by suppressing impurities/outgassing), or does the temperature itself materially affect stopping, extraction, or chemistry? This matters for how straightforward implementation would be in typical university laboratories.

Response: We thank the reviewer for this important point. In the revised manuscript, we clarify in Section 2 that cryogenic operation is not fundamentally required for stopping cells; a historic discussion of room temperature cells can be found in [50]. However, because recoils from heavy alpha decays have short stopping ranges in light buffer gas, compact gas-cell implementations operated at moderate helium pressures and even at room temperature are already sufficient for recoil stopping and extraction. In this sense, a clean room-temperature stopping cell can already provide a viable route for alpha-recoil harvesting in a typical university laboratory.

In the present work, the main advantage of the cryogenic stopping-cell operation is the very high gas purity it enables, through suppression of outgassing and impurity-related charge exchange. This is particularly beneficial for preserving higher ionic charge states and minimizing parasitic chemistry, both of which were important for the present first demonstration of controlled ion--molecule reactions with radioactive recoil ions. We believe the revised text now makes this distinction sufficiently clear.

* I recommend stating the source activity explicitly in the main text, and giving representative observed count rates for Ra in the different charge states. This would enable an efficiency estimate: what fraction of recoil daughters are stopped, transported, and ultimately detected? Where does the dominant loss occur, and is there a clear path to improving it?

Response:

We thank the reviewer for this helpful suggestion. In the revised manuscript, we now state the source activity explicitly in the main text and provide representative detected count rates at the MR-TOF-MS. Specifically, we now note that the installed $^{228}$Th source had a nominal activity of about 7kBq, and that representative detected rates at the MR-TOF-MS were about 5-15 ions $s^{-1}$, depending on the tuning conditions.

Regarding the charge-state distribution, Rn and Tl were observed only as singly charged ions after extraction from the stopping cell, whereas Ra is predominantly extracted in the 2+ charge state. From previous characterization measurements under comparable stopping-cell conditions, the extracted Ra beam is approximately 80% - $^{224}Ra^{2+}$ and 20% - $^{224}Ra^{+}$, while Po and Pb are extracted in roughly comparable fractions of 1+ and 2+.

Overall, we estimate a geometric factor of about 40% for emission of recoil products into the stopping-cell gas, near-unity stopping efficiency for those recoils entering the gas volume, and a stopping-cell extraction efficiency of order 20-30%, depending on the species, based on independent detector measurements within the setup. The dominant additional loss occurs downstream of the reaction region during transport into the MR-TOF-MS and final detection; based on the observed extracted-beam and detected rates, the effective efficiency of this stage is estimated to be only of order a few per cent in the present configuration.

This also identifies a clear path to improving the detected yields in future implementations, namely further optimization of downstream ion optics for transport into the MR-TOF-MS. In the present measurements, this transport must accommodate a very broad mass-to-charge range, e.g. from a/q=112 ($^{224}Ra^{2+}$) to a/q=243 ($^{224}RaF^{+}$) in order to study the reaction kinematics, so the ion-optical settings necessarily represent a compromise. In a dedicated production scenario, by contrast, the downstream transport could be optimized specifically for a single species of interest, for example $^{224}RaF^{+}$, rather than for simultaneous transmission across such a broad mass-to-charge range.

* Closely related: a short discussion of realistic scaling prospects would be valuable for readers assessing what future measurements might be feasible. For example, recoil sources cannot be arbitrarily thick; additional limitations may arise from space-charge effects or ion transport saturation.

Response:
We thank the reviewer for this helpful suggestion. In the revised manuscript, we have added a short discussion of realistic scaling prospects in the Conclusions/Outlook section. We now clarify that scaling is not linear with source thickness, because only recoils produced sufficiently close to the source surface can escape into the stopping gas. We also note that source activities in the range of about 300 kBq have been used in the past and appear straight forward by increasing the source area rather than the source thickness, whereas activities approaching the 10 – 100 MBq level would generally require very large stopping volume (source surface area) and hence a dedicated large offline gas-cell implementation. We further note that, at higher total ion rates, additional limitations may arise from space-charge effects and transport saturation in the RFQ and accumulation regions.

* There appears to be an inconsistency in the quoted $SF_6$ pressure: the text cites ~$8\times10^{-5}$ mbar for some measurements, whereas the table caption gives $9(4)\times10^{-3}$ mbar. Perhaps this refers to different conditions during those measurements. In any case, how are these pressures determined, and how is the quoted uncertainty obtained? At present the uncertainty appears somewhat ad hoc, yet it is central to the comparison with other alkaline-earth systems. I would also recommend the authors provide more detail on the fitting approach (least squares? likelihood fits?) and that the explicit rate equation model is also given in the methods section, for completeness.

Response:
We thank the reviewer for pointing out this ambiguity. The two quoted pressures refer to different gases in the reaction region and were not sufficiently distinguished in the original manuscript. The value $9(4)*10^{-3}$ mbar refers to the helium buffer-gas pressure used for collisional cooling and transport, whereas the reactive-gas partial pressure relevant for the kinetics measurements was $P(SF_6) = 9(6)*10^{-5}$ mbar. In the revised manuscript, we now explicitly separate P{He} and P{$SF_6$} in the Results, Methods, and the relevant table caption.

We have also clarified how the $SF_6$ pressure was determined. In the revised Methods, we now state that the $SF_6$ partial pressure was inferred from the calibrated needle-valve setting and the measured pressure increase, monitored with the vacuum gauge in the reaction region, under low-base-pressure conditions. We further explain that the comparatively large relative uncertainty in P{$SF_6$} arises from the indirect nature of this estimate and dominates the uncertainty budget of the absolute bimolecular rate coefficients. To make this more transparent, we now state explicitly that the dominant uncertainty contributions are the relative uncertainty in P{$SF_6$} and the fit uncertainty, and that these were combined in quadrature to obtain the quoted overall uncertainty.

Regarding the fitting procedure, we agree that this should be stated more explicitly. In the revised manuscript, we now specify that the time constants were obtained by least-squares minimization. We also added the effective first-order kinetic model to the Methods section for completeness.

We hope that these revisions resolve the ambiguity in the pressure values, clarify the uncertainty treatment, and make the kinetic analysis procedure sufficiently explicit.

Reviewer #2 (Remarks to the Author):

Referee report.
Nature Communications manuscript NCOMMS-25-102894

A pathway towards decentralized studies of radioactive post-lead elements and their applications in beyond standard model physics
P.M. Reiter et al

The manuscript by Reiter and colleagues presents an efficient method to harvest long-lived radioactive isotopes for research without the general requirement of a large-scale accelerator facility. Of particular interest in this work is the production of radioactive molecules, which have undergone increasing interest from different fields in recent years (e.g., for fundamental symmetries, astrophysics, nuclear structure and quantum chemistry). This interest in part has been due to advances in the ability to create, cool and control molecules, and to wider opportunities for production of radioactive molecules at RIB facilities.
The approach presented here would potentially be accessible for smaller (university-scale) laboratories. In brief, the authors employ a cryogenic gas-filled stopping cell as an environment for harvesting radioactive ions from a 228Th source. A combination of DC and RF fields are used to extract the ions from the cell into a radiofrequency quadrupole beam line. Operation of this device as a low-resolution mass separator allows for charge state selection of the ions which, in a high-purity environment, typically survive as singly- or doubly charged ions (albeit higher charge states in some cases are possible). Elements in the beam line are tuned to accumulate ions which are then exposed to controlled trace amounts of reactive gas, in this case SF6, to explore fluorination reactions with the different radioactive decay daughters (Ra, Po and Pb ions) extracted from the gas cell.
As the authors discuss, the present work focuses on the formation of RaF given its importance as a candidate for searches of physics beyond the standard model. To quantify the underlying gas-phase reaction rates between Ra+/2+ and SF6, as well as with other nuclei in the decay chain, sensitive identification of the ion species is necessary. The chosen method is high-resolution mass spectrometry in which unambiguous mass identification is demonstrated through resulting time-of-flight mass spectra. Reiter and colleagues use the mass fingerprint to study the evolution of the different species under different experimental conditions: number of revolutions in the MR-TOF device, selection of different charge states and the introduction – or otherwise – of SF6 gas. By identifying the relevant species and the relative intensities of the mass peaks, the authors demonstrate a clear sensitivity to reaction processes as a function of accumulation time in the RFQ beam line, with reaction time constants extracted from simple exponential decay and/or growth curves. This information is combined with the measured SF6 concentration to determine fluoridation reaction rates, with data for Ra obtained for the first time. The authors compare their values with literature measurements of lighter alkaline earth metal ions, with the new results found to be in

general agreement.
Finally, the experimental observations are interpreted using quantum-chemistry calculations, starting with restricted open shell Hartree-Fock calculations as a reference, and subsequent unrestricted coupled-cluster computations. These methods were assessed by calculating ionization energies, electron affinities and dissociation energies for the different atoms and molecules of interest (expounded upon in the Methods), and benchmarking against (limited) experimental and theoretical values from literature. The experimentally observed reaction pathways as elucidated from the mass spectra are then compared with the computed reaction energies which may be endothermic or exothermic. Those predicted to be exothermic in nature were observed in the mass spectra.
In summary, the article is well written and accessible to the general reader. The work has been put into context with efforts to develop new platforms for fundamental physics studies, as well as with the long-standing field of gas-phase ion chemistry. The experimental methodology is robust and sufficiently well explained, with suitable use of figures and tables. The analysis of the mass spectra and the analytical approach to determine reaction rates is valid. The additional inclusion of quantum-chemistry calculations provides added value to the experimental data, supporting the understanding of the reaction pathways open to certain elements and molecules, and validating the non-observation of others which can be explained through the computed reaction energies. Given the novelty of the methodologies with the clear potential for future harvesting of other long-lived actinide species as well as further gas-phase ion chemistry studies (both of other elements as well as trace gases), I believe this article is suitable for the Nature Communications audience. I have only a few minor comments for the author's consideration.

We thank Reviewer #2 for the positive assessment of the manuscript and for the many careful and constructive suggestions, which helped us improve the clarity, presentation, and completeness of the paper.

1. Given the use of the 228Th source in this work and the variety of accessible decay products, it would be helpful to include a figure showing the basic decay scheme, decay mode and half-lives. Such a figure could be considered for the Methods section 6.1. This also gives context for the reader to understand that all species are extracted from the gas cell within the extraction time of the cell – which I don't recall being explicitly mentioned however could be included in the Methods.
Response: We thank the reviewer for this helpful suggestion. In the revised manuscript, we have added a short explanatory paragraph in the Methods section that summarizes the relevant decay sequence and decay modes of the $^{228}$Th chain and clarifies that, in the present recoil-harvesting scheme, recoil products are made accessible when an alpha decay imparts sufficient recoil energy for the product nucleus to leave the source surface and enter the stopping-cell gas. To highlight the decay sequence in the manuscript without introducing an additional display item beyond the Nature Communications limit, we include the decay scheme directly in the Methods section as text. We also now state explicitly that extraction from the stopping cell occurs on the ~ 60 ms ms timescale under the present operating conditions; the shortest-lived recoil daughter studied here is $^{216}$Po with $t_{1/2}$~ 150  ms, so all recoil products are removed from the cell well within their respective half-lives. For completeness, we additionally cite Miskun et al. (2019), which provides a dedicated graphical decay-scheme representation.

2. Although the authors stress several times that the methods employed allow for future "decentralized studies" without the need for accelerators and/or reactors, one might argue that the cryogenic stopping cell employed in this work is perhaps not the simplest of devices for a university

laboratory. Given the small recoil energy in alpha decay of heavy elements, it would be worthwhile adding a sentence at the end of Section 2 to stress that smaller scale gas cells would be applicable to realize extraction of recoil ions as described in this work.
Response: We thank the reviewer for this helpful suggestion. We agree that the cryogenic stopping cell used in the present work is not the only possible implementation of the recoil-harvesting concept. In the revised manuscript, we now clarify in Section 2 that alpha-decay recoils from heavy nuclei can in principle be stopped and extracted in more compact gas-cell geometries operated at moderate helium pressure and even at room temperature. We also note that, for alpha-recoil harvesting, the reduced pressure, short stopping path, and fast extraction times relax the gas-purity requirements and associated operational challenges compared with large stopping cells used for energetic reaction products.

We chose to develop and test the present scheme at the FRS Ion Catcher because it provided an existing, well-characterized platform with excellent gas purity, established ion transport, and high charge-state survival, which was particularly advantageous for a first demonstration of controlled ion--molecule chemistry with radioactive recoil ions. In support of this, the manuscript shows that in the absence of $SF_6$ gas, $^{224}Ra^{2+}$ ions can be stored in the reaction region for up to 0.5s with only small losses (Methods, Fig.7), indicating sufficiently low impurity-driven charge exchange under the present operating conditions at the FRS Ion Catcher. In addition, the MR-TOF-MS part of the FRS Ion Catcher enables direct, broadband, and high-resolution verification of both the extracted recoil ions and the produced molecular species, which was essential for the unambiguous identification of reaction products in the present work.

At the same time, the underlying harvesting concept is not restricted to this specific large cryogenic implementation. The updated manuscript makes this clearer now.

3. Abstract line 61, "…further supported by…"
Response: We thank the reviewer for catching this wording issue. The sentence has been corrected in the Abstract.

4. Figure 1, correct "accumulation" in the label pointing towards the reaction region.
Response: We thank the reviewer for noting this typographical error. The label in Figure 1 has been corrected.

5. I suggest reordering Figures 2 and 3, given Fig. 3 is referenced first in the text.
Response: We thank the reviewer for this suggestion. We have reordered the figures so that they now appear in the same order as they are first cited in the text.

6. A sentence or appropriate reference could be added to indicate what kind of peak fit is used when extracting the intensities of the ion species which are used in the gas-phase chemistry analysis.
Response: We thank the reviewer for this suggestion. We have added a sentence to the Methods section clarifying how the ion-species intensities used in the gas-phase chemistry analysis were obtained. Specifically, peak identities and masses were validated using the standard gaussian peak-shape description of the MR-TOF-MS response, whereas the relative intensities used for the kinetics analysis were determined directly from the integrated number of single-ion events within narrow time-of-flight windows, since all relevant peaks were well separated.

7. Page 10, line 445, "…or release of the reactions studies."
Response: We thank the reviewer for noting this typographical error. The sentence has been corrected.

8. Figure 5 and Table 2. I might be mistaken, but can you clarify the singly charged reaction pathway between MF+ and H2O? In Fig. 5 it appears that for Ra and Po the energy for both is negative (see right hand side of the figure), while in the last row of Table 2 they are positive (values 0.45 eV and 0.323 eV, respectively).

Response:
We thank the reviewer for identifying this ambiguity. The issue arose because the left- and right-hand sides of Fig. 5 correspond to different reaction path and were referenced differently in the original presentation. On the left-hand side, the reactions $M^{n+} + SF_6 \rightarrow MF^+ + SF_5^{(n-1)+}$ for M= Ra, Pb, Po were plotted with the energies relative to the exit channel, whereas on the right-hand side the reactions $MF^+ + H_2O \rightarrow MOH^+ + HF$ were plotted with the energies normalized to the entrance channel. This was done to highlight the envisioned two-step process, but the reference change was not made sufficiently explicit and could therefore be misread. As a result, the axis direction on the right-hand side was easily misread. We have revised Fig. 5 to clarify the reference convention and to remove this ambiguity.

9. Page 12, lines 512-513, "…as a potential loss channel, for example HF formation…" Line 532, "Despite the larger uncertainty…"
Response: We thank the reviewer for these wording suggestions. The relevant sentences have been revised for grammatical correctness and clarity.

10. Table 3. It was not immediately clear to me why m(Ca) = 44u was used, as mentioned in the caption. Naively perhaps I would think 40 u would have been chosen.
Response: We thank the reviewer for pointing this out. We have clarified in the table caption that a mass of 44 u was used for Ca in order to remain consistent with the literature comparison adopted here, which was reported for $^{44}$Ca because also the actual experiment was performed with this isotope. Using 40 u instead would only slightly change the numerical Langevin capture rate and does not affect the conclusions.

11. Reference 31 seems to be missing information. Is this a thesis? If so, consider adding the relevant institution.
Response: We thank the reviewer for noting this issue. The previous reference has been updated and replaced by the corresponding published article: Athanasakis-Kaklamanakis, M., Au, M., Kyuberis, A. et al. Laser spectroscopy and CP-violation sensitivity of actinium monofluoride. Nature 648, 562–568 (2025).

Reviewer #3 (Remarks to the Author):

The manuscript "A pathway towards decentralized studies of radioactive post-lead elements and their applications in beyond standard model physics" demonstrates an important step toward the

expansion of off-line capabilities in the study of radioactive molecules, and provides measurements which are of interest on their own. Given the rapid increase in interest in the precision study of radioactive atoms and molecules, further tools which enable their study are likely to be widely adopted, especially outside of the few large facilities with direct access to radioactive beams. Both the methods and results appear to be sound, are likely of broad interest, and will likely be impactful to a wide community. I believe the manuscript to be suitable for publication in Nature Communications, though I recommend additional discussion about other approaches to decentralized studies.

As written, a non-expert reader would probably conclude that this is the first demonstration of a decentralized approach, though that is not the case. There are many such avenues being pursued, but two relevant ones have been experimentally demonstrated: trapped radium atomic and molecular ions at UCSB, and cryogenically-cooled neutral radium molecules at Caltech. The manuscript offers some distinct advantages over these other two methods, but there are also some important similarities.

The UCSB group has demonstrated work with multiple molecular species formed through controlled chemical reactions [reference 61] and multiple radium isotopes, including two short-lived isotopes Ra-224 [reference 62] and Ra-225 [2407.14721, unreferenced]. The UCSB Ra-224 work used decays from Th-228, as in this manuscript. However, the manuscript work uses a gas cell, which makes the approach applicable to a wider range of studies outside of ion trapping, to which the UCSB approach (as currently implemented) is largely limited.

The Caltech group has demonstrated cryogenic formation of multiple radium-containing species including polyatomics via laser-controlled chemistry, and has shown high resolution optical spectra [2508.08368, not referenced]. However, the manuscript approach does not rely on any sort of handling of the radioactive species of interest, and is therefore well-suited to short-lived species as discussed in the caption of Fig. 1.

My specific recommendation is that there be some discussion in the introduction, on the level of a few sentences, which compare and contrast with these different approaches, and perhaps a brief mention in the conclusion as well. The UCSB results are mentioned very briefly in passing, but should be discussed more deliberately; the Caltech work is not referenced. Again, these studies do not detract from the impact or novelty of the present result, as they all provide a distinct set of advantages.

**Responds to Reviewer #3**

We thank the reviewer for the positive assessment of our manuscript and for the helpful suggestion to better position the present work relative to other decentralized approaches. We agree that, in the original version, a non-expert reader could have inferred too broad a claim regarding the novelty of decentralized access to radioactive radium species.

In response, we have revised the Introduction to explicitly acknowledge complementary laboratory-scale routes that have recently emerged. In particular, we now cite recent work demonstrating thorium-generator-based in-vacuo production, trapping, and laser cooling of $^{224}Ra^+$ and $^{225}Ra^+$ ions, as well as cryogenic buffer-gas production and high-resolution spectroscopy of neutral radium-containing molecules including RaOH and RaF. We further clarify that the present work is

complementary to these efforts, rather than positioning it as the first decentralized approach in a general sense.

We also slightly revised the opening of the subsequent paragraph to make the relation to these prior efforts explicit by describing our scheme as a complementary, broadly applicable, accelerator-independent route.

We hope this revision addresses the reviewer's concern by making clear that the present manuscript introduces a distinct and complementary pathway based on recoil-ion harvesting, controlled ion--molecule reactions, and broadband high-resolution mass spectrometric identification of radioactive molecular ions, while properly acknowledging related recent progress in the field.
